# Complete and incomplete Kawasaki disease: Clinical differences and coronary artery outcome from a national prospective surveillance study in Switzerland

**DOI:** 10.3389/fped.2023.1137841

**Published:** 2023-03-20

**Authors:** S. Bressieux-Degueldre, E. Gradoux, S. Di Bernardo, N. Sekarski

**Affiliations:** ^1^ Pediatric Cardiology Unit, Department of Women-Mother-Child, Lausanne University Hospital, Lausanne, Switzerland; ^2^ Department of Women-Mother-Child, Lausanne University Hospital, Lausanne, Switzerland

**Keywords:** Kawasaki disease, outcome, complete form, incomplete form, coronary artery aneurysm

## Abstract

**Introduction:**

The aim of this national prospective surveillance study was to compare the clinical presentation, laboratory findings, treatment, and coronary artery outcome in patients with incomplete and complete Kawasaki disease (KD).

**Methods:**

Between March 2013 and February 2019, children with a diagnosis of complete and incomplete KD were reported by the Swiss Paediatric Surveillance Unit and prospectively enrolled. Clinical data, laboratory values, treatment, and echocardiographic features were collected at diagnosis and 1 year of follow-up. Data were compared between children with complete or incomplete KD.

**Results:**

A total of 351 questionnaires were registered from children with a diagnosis of KD. Of them, 219 (62.4%) children had complete KD, and 132 (37.6%) children had incomplete KD. Children with incomplete KD were younger and had a longer-lasting fever; however, there were no differences in the level of C-reactive protein. All but four children received intravenous immunoglobulin treatment, whereas 14% of children were treated with corticosteroids. Children with incomplete KD were more often treated with corticosteroids than children with incomplete KD (*p* = 0.01). At diagnosis, 39 (11.1%) patients had only coronary artery dilation and 57 (16.2%) had at least one coronary artery aneurysm. There were no differences in coronary artery involvement between the two groups. At follow-up, 273 of 294 (92.8%) patients had no coronary artery involvement, with no difference between the two groups (*p* = 0.609). The overall incidence of coronary artery aneurysms at diagnosis was 16.2%. At follow-up, most coronary artery aneurysms had regressed, and coronary artery aneurysms were present in only 5.8% of the patients. Coronary artery aneurysms were slightly more frequent in patients with incomplete KD at follow-up (*p* = 0.039) but not at diagnosis (*p* = 0.208).

**Conclusion:**

Although the clinical presentation in children with incomplete and complete KD differs, the absence of coronary artery involvement does not. The use of corticosteroids appears to be preventive against the development of coronary artery aneurysms in these patients. However, the results of this study suggest a lower rate of coronary artery aneurysm regression in patients with incomplete KD. Further studies on a larger scale are needed to assess the risk of non-regression of coronary artery aneurysms in this particular group of patients.

## Introduction

A diagnosis of Kawasaki disease (KD) is based on clinical and laboratory criteria, and no definitive diagnostic test exists. The diagnosis of incomplete KD remains challenging, and diagnostic delays often occur, especially in infants. A more severe coronary outcome in children with incomplete KD has been suggested but remains controversial (1, 2). A previous study on the epidemiology of KD in Switzerland showed that children aged under one year of age or older than eight had more echocardiographic abnormalities at diagnosis (3).

A prompt diagnosis and rapid treatment with intravenous immunoglobulin (IVIG) remain the mainstays, as they have been shown to be effective in reducing coronary artery aneurysms (CAAs) (4).

The aim of the present study was to compare the clinical presentation, laboratory findings, treatment, and mid-term coronary artery outcome in patients with complete or incomplete KD in Switzerland. We hypothesized a more severe coronary outcome for patients with incomplete KD.

## Methods

Children aged under 18 years with a diagnosis of complete or incomplete KD who were admitted between March 2013 and February 2019 in one of the 33 pediatric clinics in Switzerland and reported by the Swiss Paediatric Surveillance Unit (SPSU) were prospectively enrolled.

As for our previous study on the epidemiology of KD in Switzerland (3), an announcement was made by the physician in charge of the patient through the SPSU (www.spsu.ch), which is a surveillance system for rare pediatric diseases in hospitalized children in each of the 33 Swiss pediatric hospitals set up by the Swiss Pediatric Association and the Federal Office of Public Health. After receiving the initial anonymous announcement from the SPSU, a questionnaire was sent to the physician in charge of the patient during the hospital stay.

Demographic and clinical data were collected, including diagnostic clinical features for KD (bilateral non-purulent conjunctivitis, oral mucosal changes, cervical lymphadenopathy, skin rash, and changes in the extremities) and the duration of the fever. The results from laboratory investigations were recorded, including the most abnormal values during hospitalization for C-reactive protein (CRP), albumin, hemoglobin (HBG), white blood cell count (WBC), and platelet count.

Patients with a diagnosis of KD were matched either to the “incomplete KD” or the “complete KD” group according to the American Heart Association (AHA) guidelines (5, 6).

A follow-up questionnaire was sent 1 year after diagnosis to the pediatric cardiologist listed on the initial questionnaire. Clinical symptoms were recorded during the follow-up. This included symptoms of heart failure, chest pain, and/or shortness of breath at rest or during exercise.

The data from the echocardiography indicating a presence or absence of coronary artery dilation and/or aneurysms were recorded at the time of diagnosis and during the follow-up. In the case of coronary artery involvement and data availability, coronary artery dimensions for the right coronary and the left coronary artery (expressed in mm) were recorded and z-scores were calculated (7). Coronary artery dilation and the degree of the coronary aneurysm were defined according to the AHA guidelines (6).

Initial treatments with IVIG and doses of acetylsalicylic acid (ASA), together with any additional immunomodulatory therapy (e.g., corticosteroids, anakinra), were recorded. Medical treatments during the follow-up were collected.

Demographic data, clinical symptoms and signs, laboratory values, treatments, and coronary artery outcomes at presentation were compared between the incomplete and complete KD groups.

The coronary artery outcome between the two groups was compared during the follow-up.

Data have been reported as the mean with SD or the median with interquartile range, as appropriate. The statistical significance of differences between patients with complete and incomplete KD was assessed using the Student's *t*-test for continuous variables and Pearson's chi-square test for categorical variables. A *p*-value <0.05 was considered significant.

The research protocol was approved by the Institutional Ethics Board (CER-VD). Because of the anonymous data collection, the Ethics Committee waived informed consent. The study was performed in compliance with the 1964 Helsinki Declaration and its later amendments.

## Results

Between March 2013 and February 2019, 351 questionnaires were collected from children with a diagnosis of KD. Of them, 219 (62.4%) children had complete KD, and 132 (37.6%) children had incomplete KD. In children aged under one year of age, the proportion of incomplete KD was even higher, at 54.5%. The demographic, clinical, and laboratory features of children with complete or incomplete KD at the time of diagnosis are illustrated in Table 1.

**Table 1:** Clinical and laboratory data at diagnosis.

	Incomplete KD	Complete KD	*p*-value
Age [mean (SD) in months]	34.8 (32.3)	45.0 (35.3)	0.004
Sex (male *N*, %)	81 (61%)	128 (58%)	0.787
Clinical symptoms
Rash	88/128	212/218	0.005
ADP	35/121	135/214	<0.001
Extremities	48/127	181/215	<0.001
Mucosa	78/127	210/219	<0.001
Conjunctivitis	72/122	204/219	<0.001
Duration of fever [mean (SD) in days]	9.1 (4.0), *n* = 105	7.2 (3.3), *n* = 167	<0.001
Laboratory
CRP level, g/L (mean/SD)	117.8 (77.0)	115.2 (87.5)	0.389
HBG level, g/L (mean/SD)	97.3 (18.3)	104.8 (16.3)	<0.001
Thrombocytes G/L (mean/SD)	407.9 (220.75)	344.0 (148.8)	<0.001
WBC	20.7 (22.7)	17.3 (11.3)	<0.001
Albumin	30.0 (6.2)	32.2 (9.4)	0.030

KD, Kawasaki disease; ADP, adenopathy; CRP, C-reactive protein; HBG, hemoglobin; WBC, white blood cell count.

Continuous variables are expressed in mean (SD). Categorical variables are expressed in *N* (%).

The mean age at diagnosis was 3.4 ± 1.2 years. Children with an incomplete KD were younger and had a longer-lasting fever. The typical clinical symptoms were significantly less frequent in children with incomplete KD, with rash being the most common symptom in both groups. There were no differences in inflammatory markers, such as CRP levels, but all other recorded laboratory parameters (hemoglobin, thrombocytes, white blood cells, and albumin) did differ (Table 1). For the latter laboratory parameters, the children with incomplete KD had more abnormal levels compared to the children with complete KD.

The medical therapies at the time of diagnosis and 1 year of follow-up are illustrated in Table 2. All but four (98.6%) children received IVIG and aspirin treatment at the time of diagnosis, with no differences between the two groups. Of them, one child was diagnosed retrospectively with incomplete KD due to axillary artery aneurysms more than 1 year after the acute disease, two children had a late diagnosis of KD and were already afebrile at the time of diagnosis, and in one child, no details were given regarding the reason for no treatment. Interestingly, these last three children did present with a complete form of KD. All children received an IVIG dose of 2 g/kg, whereas the initial aspirin dose varied between 50 and 80 mg/kg in patients with incomplete KD. Approximately one-fifth of the patients received a second dose of IVIG; however, there were no differences between the children with complete and incomplete KD. Of all the children, 14% were treated with corticosteroids. Children with incomplete KD were more often treated with corticosteroids compared to children with incomplete KD (*p* = 0.01).

**Table 2:** Medical therapy at diagnosis and at follow-up.

	Incomplete KD	Complete KD	*p*-value
Treatment at baseline			
Dose of IVIG in g/kg	2	2	
IVIG (initial treatment), *N* (%)	131 (99.2)	216 (98.6)	0.95
2^nd^ dose of IVIG, nb of patients (%)	29/125 (23.2) 29/132 (22)	43/207 (20.7) 43/219 (19.6)	0.60 0.64
Corticosteroids, nb of patients (%)	23/132 (17.4)	28/219 (12.8)	0.01
Aspirin treatment at diagnosis, *N* (%)	131 (99.2)	216 (98.6)	0.95
Dose of Aspirin at diagnosis in mg/kg (Median/IQR)	80 (50-80)	80	
- VKA (*n*/%) - Clopidogrel (*n*/%) - Other (*n*/%)	7 (5.3) 2 (1.5) 0 (0)	1 (0.4) 2 (0.9) 1 (0.4)*	
Treatment at FU			
- Aspirin (*n*/%) - VKA (*n*/%) - Clopidogrel (*n*/%) - Betablocker (*n*/%)	20 (15) 4 (3.0) 0 0	16 (7.3) 2 (0.9) 1 (0.5) 1 (0.5)	

IVIG, intravenous immunoglobulins; IQR, interquartile range; VKA, Vitamin K antagonist; FU, follow up.

* Enalapril.

Additional anticoagulation therapy with vitamin K antagonists or antiplatelet therapy with clopidogrel was added in 12 patients.

The follow-up questionnaires of 294 patients were reviewed. The remaining 56 patients were either lost to follow-up or the questionnaire was not returned. One child died during the acute phase of KD.

The mean time from diagnosis to follow-up was 10.1 ± 5.5 months. This is explained by the fact that, although the questionnaire was sent out 1 year after the acute illness, some cardiologists in charge of the patients did see them earlier for follow-up and discharged them earlier. At follow-up, aspirin was continued in 36 (12.2%) patients. Six patients received therapy with vitamin K antagonists. Two patients had additional therapy (one patient had clopidogrel, and one had beta-blockers).

At the time of follow-up, all patients were free of cardiovascular symptoms.

In total, 255 (72.6%) children had no coronary artery involvement at the time of diagnosis. Of the 96 (27.3%) children with coronary artery involvement, 39 (11.1%) patients had only coronary artery dilation, and 57 (16.2%) had at least one CAA on echocardiography. There were no differences in coronary artery involvement between both groups (Table 3). For those patients with coronary aneurysms, right coronary artery (RCA) aneurysms were more frequent in patients with incomplete KD compared with patients with complete KD (*p* = 0.038).

**Table 3:** Coronary artery involvement.

At diagnosis	Incomplete KD (*n* = 132)	Complete KD (*n* = 219)	*p*-value
No CA involvement	86 (65.1%)	169 (77.2%)	0.201
CA dilation only	20 (15.1%)	19 (8.7%)	0.080
At least one aneurysm	26 (19.7%)	31 (14.2%)	0.208
RCA dilation	17 (12.9%)	23 (10.5%)	0.514
RCA aneurysm	12 (9.1%)	8 (3.7%)	0.038
LCA dilation	37 (28.0%)	43 (19.6%)	0.111
LCA aneurysm	12 (9.1%)	9 (4.1%)	0.065
At follow-up	Incomplete KD (*n* = 111)	Complete KD (*n* = 183)	*p*-value
No CA involvement	99 (89.2%)	174 (95.1%)	0.609
CA dilation only	2 (1.8%)	3 (1.6%)	0.873
At least one aneurysm	10 (9.0%)	6 (3.3%)	0.039
RCA dilation	2 (1.8%)	0 (0%)	
RCA aneurysm	6 (5.4%)	4 (2.2%)	
LCA dilation	2 (4.5%)	4 (2.2%)	
LCA aneurysm	8 (7.2%)	3 (1.6%)	

KD, Kawasaki disease; CA, coronary artery; RCA, right coronary artery; LCA, left coronary artery.

**Figure 1:**
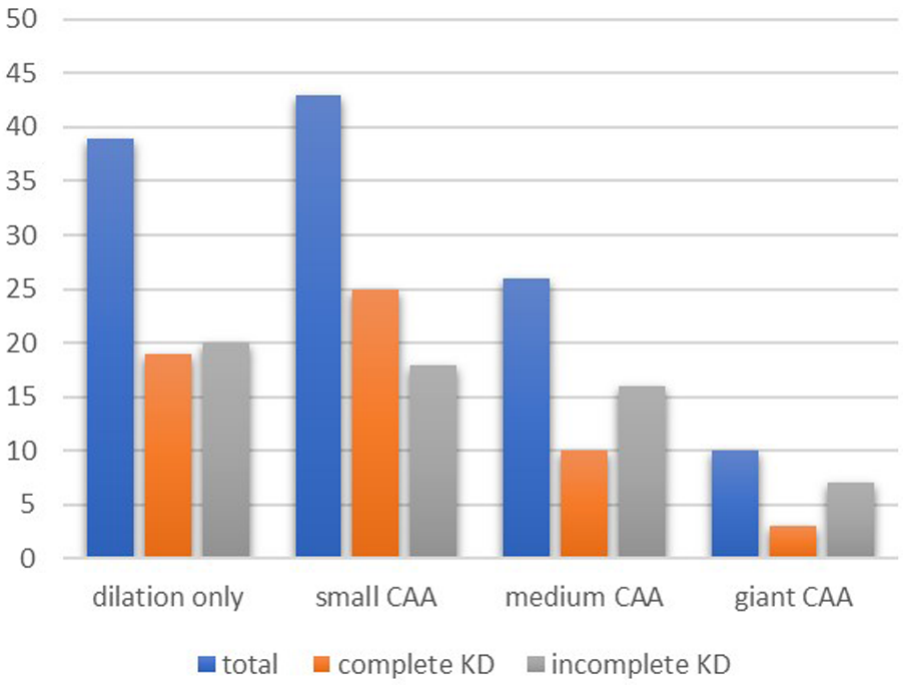
Coronary artery involvement at diagnosis. Pearson's chi-square test showed no differences between the groups for complete and incomplete KD in patients with dilation only (*p* = 0.08), small CAA (*p* = 0.91), medium CAA (*p* = 0.11), and giant CAA (*p* = 0.14). KD, Kawasaki disease; CAA, coronary artery aneurysm.

Of the 57 patients with at least one CAA, 30 (52.6%) patients had small CAAs, 18 (31.6%) patients had medium-sized CAAs, and 8 (14.0%) patients had giant CAAs. Of those patients with giant CAAs, five had incomplete KD, and three had complete KD. Figure 1 shows the distribution of the severity of CAAs between the two groups. In one patient with CAA, no data were given on the exact size; therefore, the severity of the CAA could not be assessed. Small, medium-sized, and giant CAAs were equally present in both groups (Figure 1).

**Figure 2:**
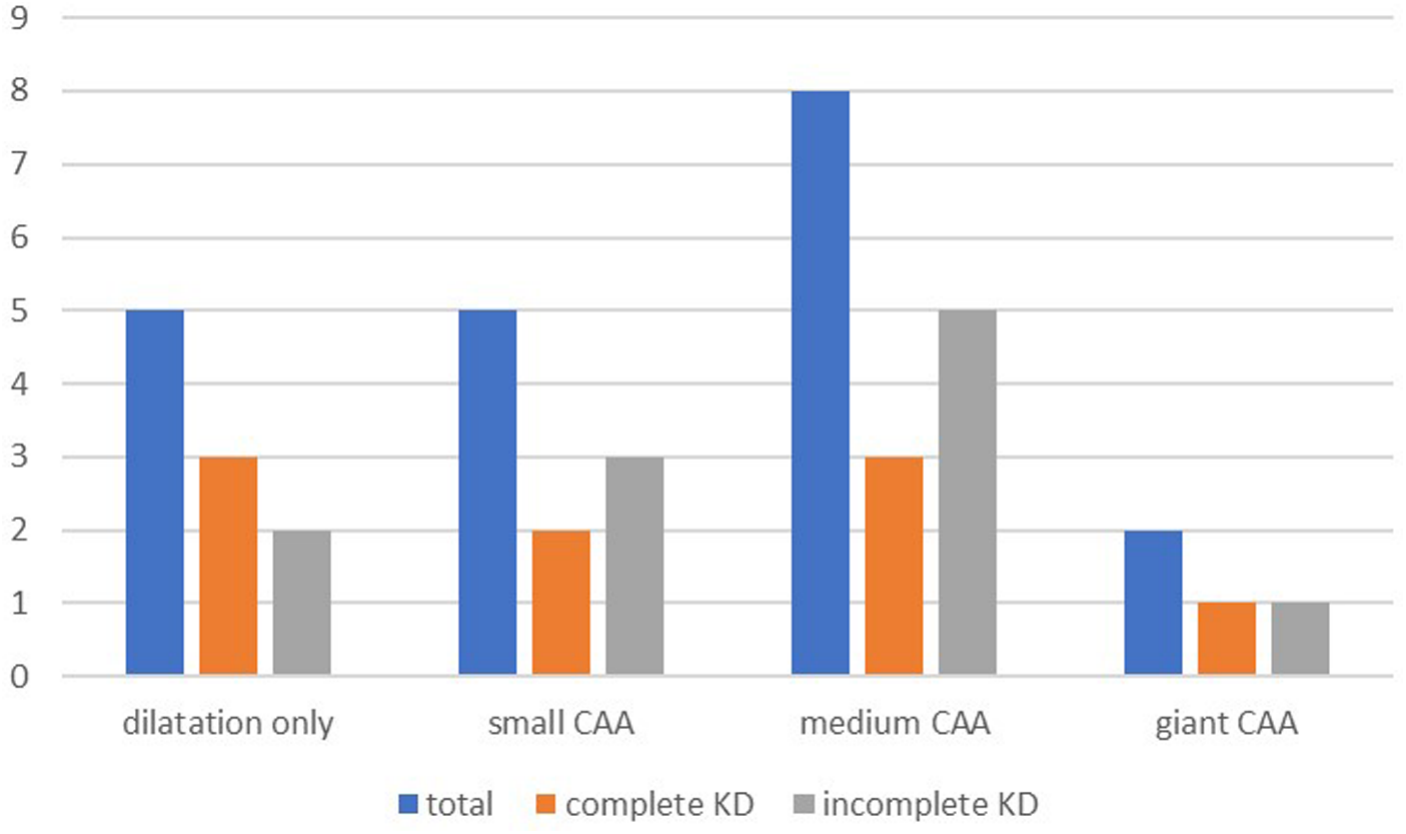
Coronary artery involvement at follow-up. Pearson's chi-square test showed no differences between the groups for complete and incomplete KD in patients with dilation only (*p* = 0.873), small CAA (*p* = 0.307), medium CAA (*p* = 0.149), and giant CAA (*p* = 0.741). KD, Kawasaki disease; CAA, coronary artery aneurysm.

At the follow-up, 273 of 294 (92.8%) patients had no coronary artery involvement, with no difference between the two groups (*p* = 0.609). Coronary artery dilation was seen in 5 (1.7%) patients and 16 (5.8%) patients had at least one coronary aneurysm. The presence of at least one CAA was slightly more prevalent in incomplete KD (*p* = 0.039). In total, five patients had small CAAs, eight had medium-sized CAAs, and two had giant CAAs at the time of follow-up. In one patient, the dimension of the CAA was not specified. Figure 2 shows coronary artery involvement at follow-up in children with complete or incomplete KD. When comparing the severity of CAAs in patients with complete or incomplete KD, no significant difference was found between the two groups (Figure 2).

## Discussion

This is the first prospective study comparing cardiac outcomes in complete or incomplete KD during acute illness and mid-term follow-up in Switzerland.

The proportion of patients with incomplete KD versus complete KD in this national prospective surveillance study is in line with recent studies (3, 8, 9), and slightly higher than in a previous retrospective single-center study in Switzerland between 1981 and 2014, where incomplete KD represented 29.5% of all patients with KD (10).

Indeed, several epidemiological studies have shown a rise in the incidence of incomplete KD over the last several years, with some reports showing that more than 40% of KD patients have incomplete KD (2, 11–12). This is thought to be due to the impact of the guidelines published in 2004, including the laboratory criteria (13); it may also reflect the lower threshold for the diagnosis and treatment of patients with suspected KD in order to avoid cardiac sequels (12).

In the present study, the age of onset was lower in patients with incomplete KD than in those with complete KD, and in patients aged under one year of age, more than half fulfilled the criteria for incomplete KD. Indeed, the proportion of patients with incomplete KD has been shown to be particularly high in patients aged younger than one (14) and even higher, up to 78.1%, in patients aged under six months (15, 16). This might be due to their immature immune systems, leading to a weaker response to vasculitis in the younger patient population (17).

Not surprisingly, classic clinical symptoms were less prevalent in children with incomplete KD in our study. This is in line with the American Heart Association algorithm (6), which defines incomplete KD in children as a fever of 5 days or more and two or three compatible criteria, or in infants as a fever lasting 7 days or more without any other explanation. Other studies also found significant differences in the clinical characteristics between complete or incomplete KD (1, 12), with children with incomplete KD having less conjunctival congestion, lymphadenopathy, and hand and foot redness (18).

One clinical difference between the two groups is the duration of fever, which was longer in the group with incomplete KD. This can be due to the delay in diagnosis in the last group, which was not evaluated in our study, and because of the definition of incomplete KD in infants (6), but also due to the higher rate of IVIG resistance. Other studies also reported a longer fever duration in incomplete KD patients (18).

Despite the longer duration of fever in incomplete KD patients, there were no differences in inflammatory markers, such as CRP levels, between the two groups. This has also been observed in another study comparing children with complete and incomplete KD (1, 18). In the present study, this might be explained by the greater use of corticosteroids in children with incomplete KD.

Incomplete KD is characterized by some but not all of the diagnostic criteria for KD (6). As a result, one can expect that the laboratory values in incomplete KD may not be as pronounced as those seen in complete KD. However, in this study, all laboratory values, except for CRP levels, were more abnormal in patients with incomplete KD. This might be explained by the fact that the most abnormal laboratory value during the hospital stay was recorded and not the baseline value at diagnosis. In a large retrospective study by Manlhiot et al. (1), most laboratory values differed between incomplete and complete KD, with incomplete KD having the most abnormal values.

In this study, patients with incomplete KD did not receive a second dose of IVIG more often; however, they were more often treated with corticosteroids. This might be due to the fact that patients with incomplete KD have a higher rate of IVIG resistance, needing, in addition to a second dose of IVIG, second-line treatments such as corticosteroids. Furthermore, patients in this group are younger, some aged under one year, making them a high-risk group for treatment with corticosteroids in addition to IVIG.

In this national prospective surveillance study, the overall incidence of CAA at diagnosis was 16.2%. Tulloh et al. (19) reported, in a prospective population survey in the UK and Ireland between 2013 and 2015, a rate of CAA of 19% and an incidence of 1.6% for giant CAAs during the acute phase, which is similar to this study.

At follow-up, most CAAs had regressed, and CAAs were present in only 5.8% of the patients.

Overall, 72.6% and 92.8% of the patients had no coronary artery involvement at diagnosis and follow-up, respectively, with no differences between patients with incomplete and complete KD, despite a longer duration of fever in the first group. CAAs were slightly more frequent in patients with incomplete KD at follow-up but not at diagnosis. This suggests that the CAA regression rate is lower in incomplete KD than in complete KD. Friedman et al. (20) found an association between a higher CAA *z*-score at diagnosis and a lack of regression. This cannot explain a lower regression rate in incomplete KD in our study, as there were no differences in the size of CAAs at diagnosis between the two groups. However, because of the small number of patients, further studies are warranted to identify any potential association between the regression rate of CAAs in incomplete and complete KD.

In Asian countries, especially in Japan, numerous risk scores, including Kobayashi et al. (21), Egami et al. (22), and Sano et al. (23), have been developed to estimate IVIG resistance and the increased risk of cardiac complications. However, no score has been validated in North American and European populations (24, 25).

Previous studies in non-Asian countries have been contradictory regarding the risk of coronary artery involvement in incomplete versus complete KD. A retrospective study by Manlhiot et al. (1) found no differences between children with incomplete and complete KD. Davidson et al. (26) identified from a retrospective study in South Australia that children with incomplete KD were more likely to develop CAAs. However, both were retrospective studies, and the mid-term outcome was not analyzed. In a population-based study between 2004 and 2014 in Sweden, Mossberg et al. (27) described a much higher rate of CAA (31%) during the acute phase and an association between CAA and incomplete KD, with an incidence of CAA of 46.7% in this group. However, about one-quarter of patients with incomplete KD were not treated within 10 days with IVIG. In the present study, the time from the first day of fever to treatment with IVIG was not recorded; however, the mean duration of fever was 9 days in patients with incomplete KD. The distribution of the size of the CAAs was similar to the present study, with more than half of the patients having small CAAs.

In this study, the use of corticosteroids was higher in patients with incomplete KD. This could explain the lack of difference in inflammatory markers and coronary artery involvement between the two groups despite the longer duration of fever in children with incomplete KD. Indeed, steroids have been shown to reduce the risk of coronary artery development in addition to IVIG in KD, especially when given as an initial treatment (28). Some authors recommend the use of corticosteroids as initial therapy in patients with a high risk for IVIG resistance, for example, in children aged under 1 year (29, 30).

A high index of suspicion and prompt treatment with IVIG and, when indicated, corticosteroids are crucial to reducing the risk of coronary artery involvement.

### Limitations

The present study has some limitations. First, several clinical and paraclinical data items were not completed in the questionnaire. The echocardiographic data from the left coronary artery were recorded, but the questionnaire did not specify the branches. If not specified by the treating cardiologist, we used the *z*-scores for the left main coronary artery. This might underestimate some results in terms of the severity of the CAAs of the left coronary artery. Furthermore, a number of patients were lost to follow-up. Second, during the study period, the international guidelines for the diagnosis and management of KD changed in 2017. This might have led to changes in clinical practice in the different hospitals involved in the study.

## Conclusion

Although the clinical presentation in children with incomplete and complete KD is different, especially in the duration of fever, freedom from coronary artery involvement did not differ in this nationwide prospective surveillance study in Switzerland. The use of corticosteroids appears to be protective against the development of CAAs in this patient population. However, the results of this study suggest a lower rate of CAA regression in patients with incomplete KD. Further studies on a larger scale are needed to assess the risk of non-regression of CAA in this particular group of patients.

## Data Availability

The raw data supporting the conclusions of this article will be made available by the authors upon reasonable request.
